# Next-generation sequencing of the soil nematode community enables the sustainability of banana plantations to be monitored

**DOI:** 10.1016/j.apsoil.2021.103999

**Published:** 2021-10

**Authors:** Christopher A. Bell, Josephine Namaganda, Peter E. Urwin, Howard J. Atkinson

**Affiliations:** a Centre for Plant Sciences, School of Biology, University of Leeds, Leeds, UK; b National Agriculture Research Laboratories, Kampala, Uganda

**Keywords:** Next-generation sequencing, Nematode soil community, Soil quality, Sustainable production, Banana

## Abstract

Uganda faces a considerable challenge to match its food production to an annual population growth rate of 3%. Cooking bananas are the country's most produced staple crop but the annual national harvest is not increasing. The crop grows on infertile soils that are normally fertilised organically and often susceptible to erosion. Soil nematodes are well-established as bioindicators of soil quality that can support environmental monitoring and assessment of the sustainability of agricultural systems. These invertebrates are a highly ranked indicator of biodiversity with molecular approaches available. Consequently, we have applied next-generation DNA sequencing of soil nematodes to evaluate soil quality of Ugandan banana plantations. The aim is to establish a method for constructing an aspect of an environmental biosafety dossier with the future aim of assessing the impact of transgenic crops and improving current cropping systems. The soil samples did not differ significantly in any of the measured soil chemistry factors, soil texture or percentage of organic matter. Thirty taxons of soil nematodes other than the plant parasites were recovered from soil supporting nine banana plantations plus three each from coffee and banana-coffee interplants from East and West Uganda. Cluster analysis correctly allocated each plantation to the crop/intercrop being grown when based on the abundance of taxa rather than taxa presence or absence. This indicates that the host has considerable effects on the abundance of specific nematode species within the soil. Overall, nematodes were more abundant in soil from coffee plantations than from banana-coffee interplants with the lowest values being from fields supporting just banana. Only the basal and trophic diversity indices and the percentage of nematodes that are rapid colonisers varied between the three plantation types. The soil of all fifteen plantations can be classified as having a mature soil web condition with low physical disturbance, limited chemical stressors, moderately high nutrient enrichment and balanced decomposition channels.

## 1 Introduction

The current population of Uganda is 35 million but it is expected to rise 3% annually to 100 million by 2050. The proportion of the population that suffers from hunger is declining but in 2017 Uganda was still among the 20 countries with the highest prevalence of undernutrition, with 29% of children under the age of five stunted (MAAIF 2015/6 report). Bananas (particularly those that are cooked) are the most produced food crop in Uganda (FAOSTAT, 2020). As consumed bananas deliver the most carbohydrate per unit area of harvest of all crops grown in that country but the area grown and yield/ha have declined by 10% and 6% respectively over the same period (FAOSTAT, 2020). Many decreases in production are related to soil quality and poor crop management (van Asten et al., 2003) and soil moisture deficits resulting from prolonged dry spells (MAAIF 2015/6). Banana yields in Uganda are only 7–43% of the potential harvest (van Asten et al., 2003). Much of this variation and short-fall results from complex interactions mediated through the soil environment. The factors include soil moisture levels of this rainfed crop, dependence on organic mulches and stover rather than inorganic fertilisers (Mukuve and Fenner, 2015) and biotic stresses from soil organisms. For instance, soil-borne plant parasitic nematodes frequently cause yield losses of over 50% (Roderick et al., 2012; Tripathi et al., 2015).

Sterility of banana, the lack of cross-fertile wild relatives and clonal propagation of banana all contribute to emphasis on transgenic traits to counter slow progress in improvement by conventional plant breeding. Examples include control of *Xanthomonas* wilt disease (Tripathi et al., 2014), nematode pests (Tripathi et al., 2015) and improvement in pro-vitamin A content (Paul et al., 2018). National regulators are likely to require an environmental biosafety dossier to consider before determining if uptake by growers should be authorised. One aim of this work is to contribute approaches and base-line data to assist future assessments of any impact of transgenic crops on soil quality at multi-location trials. This would support development of a dossier on the environmental biosafety of such plants.

Stewardship of current agricultural soils is essential for sustained human prosperity (Amundson et al., 2015). It is predicted that soil erosion losses from either banana-coffee interplants or banana-only plantations are only 50% and 33% respectively of the possible 93 t/ha/year lost from land growing annual crops in Uganda (Lufafa et al., 2003). This highlights differences in the relative impact of various cropping systems. The soil quality of agricultural land in Uganda varies considerably with the locality and by land-use type (Wortmann and Kaizzi, 1998). Banana is frequently grown on highly weathered soils with a low inherent fertility (van Asten et al., 2003) but often receives a disproportionate share of stover from other crops in addition to plant material from its harvest when near homesteads. This favours a neutral nutrient balance to the detriment of several annual crops on more distant hillsides (Wortmann and Kaizzi, 1998; Briggs and Twomlow, 2002). However, growers consider declining banana yields in Uganda, and specifically in the Lake Victoria Crescent, is partly due to soil fertility depletion (Bekunda, 1999).

Maintaining soil quality is important for the productivity of crops including banana and nematodes are useful bioindicators of a soil's status. Soil nematodes occupy pivotal roles in processing organic nutrients and the control of soil microorganism populations based on a range of feeding guilds as bacterivores, fungivores, herbivores, omnivores and predators (van den Hoogen et al., 2019). In contrast to bacteria, nematode populations are relatively stable in response to changes in moisture and temperature and they are not subject to short-term nutrient flushes but respond to land management changes in predictable ways (Bongers, 1990; Ingham, 2000; Zhao and Neher, 2013). Soil nematodes are a highly ranked indicator of biodiversity for which molecular approaches can be applied (Stone et al., 2016). These nematodes are responsive to natural processes such as root death, rhizodeposition and agricultural activities including soil cultivation and the addition of inorganic or organic fertilisers (Ferris et al., 2001; Fiscus and Neher, 2002; Ferris and Bongers, 2006; Sánchez-Moreno et al., 2009). A wide range of ecological indices have been applied to soil nematode communities (Li et al., 2016). One commonly adopted approach to nematode faunal analysis subdivides those contributing to soil quality by feeding guild and life history characteristics in terms of a colonizer-persister scale. This enables the level of enrichment, disturbance, decomposition channel, C:N ratio and food web condition to be defined (Ferris et al., 2001).

The central role of nematodes in the soil food web and their linkage to ecological processes offers a tool for testing ecological hypotheses and understanding biological mechanisms in soil including the sustainability of agricultural systems and environmental monitoring (Neher, 2010). Uganda and many other countries are unlikely to have sufficient expertise to analyse soil nematode communities frequently when identification relies on time consuming morphological identification. This limitation has been initially overcome by basing assessments on a molecular bar-coding approach centred on specific PCR primer pairs to identify the range of taxons present in a sample (Floyd et al., 2002). This approach was applied to determine the impact of a transgenic crop on soil quality (Green et al., 2012). Further enhancements to this method have involved next-generation sequencing of the variable sequences generated from PCR with a universal primer on a pool of nematodes. Established databases can then be referenced to annotate the sample barcodes with taxonomic classifications (Porazinska et al., 2009; Sapkota and Nicolaisen, 2015; Holovachov et al., 2017; Waeyenberge et al., 2019). These rapid assessments can be achieved remotely and even internationally as in the current work after only limited sample preparation.

We have used a high throughput approach to assess its suitability to determine soil quality differences among Ugandan banana plantations relative to when two other perennial crop selections (banana-coffee interplants and coffee only) are grown. The work establishes a high throughput, information rich approach to assess the sustainability of banana production with the potential to support increased productivity of smallholder agriculture in Uganda and elsewhere.

## 2 Materials and methods

### 2.1 Field sampling and nematode extraction

Fifteen soil samples were collected from 11 fields where banana (9 samples), coffee (3 samples) and banana-coffee (3 samples) were the dominant plants (Table 1). The western and eastern sampled regions were 250 km apart but the fields within the villages of these two regions were all within 25 km of each other. Three soil samples were taken from each field and pooled. The samples were taken on 14/06/19 (West) and 17/06/19 (East). Collection was shortly after the end of the rainy season that spans from March to June. This facilitates sample recovery while the soil remains damp. Precipitation in May 2019 was at least 200 mm across the areas sampled (UNMA, 2019). These samples were air-freighted swiftly to Leeds, UK, where nematodes were extracted from 100 g of the fresh soil using a standard approach (the tray method, see Southey, 1986). The extracted nematodes were collected after 24 h on a 25 μm sieve and concentrated by centrifugation at 3000*g* for 3 min or by leaving to settle overnight.

**Table 1:** Location of sample sites, years since last planting, selected soil characteristics and soil nematode population density.

Field	Crop	Village	Parish; region^a^	Years post-planting	Soil texture^b^	pH	Organic matter (%)	Nematodes 100 g^−1^ soil
1	Banana	Lukese	1	2	SCL	6.1	4.4	330
2	Coffee	Lukese	1	3.5	SCL	6.2	4.1	1100
3	Banana	Lukese	1	6	SCL	6.4	5.0	301
4	Banana	Kitaweera	1	3	SCL	6.6	5.2	210
4	Coffee	Kitaweera	1	5	SCL	6.9	4.6	900
5	Banana	Kitaweera	2	7	CL	6.6	4.4	400
6	Banana	Mauta	1	6	ZC	6.5	8.3	380
7	Coffee	Butemula	1	10	C	6.6	5.4	680
7	Banana-coffee	Butemula	3	3	C	6.3	8.5	406
8	Banana	Nakabango	3	2.5	C	6.2	8.2	308
9	Banana	Nakabango	3	5	C	6.5	5.3	200
9	Banana-coffee	Nakabango	3	5	C	6.1	8.0	804
10	Banana	Lwanda	3	2	CL	6.7	9.5	550
10	Banana-coffee	Lwanda	3	1	CL	6.3	4.8	450
11	Banana	Nsuube	4	2	C	6.8	7.9	350

a Parish and region of Uganda: 1; Kasambya (West), 2; Buwekula (West) 3; Namulesa (East); 4, Nawargoma (East).

b Soil Texture: C, Clay, ZC, Silty Clay; CL, Clay Loam; SCL Sandy Clay Loam; ZL, Sandy Loam.

### 2.2 Soil analysis

An aliquot of each soil sample was sent to NRM Laboratories Ltd., Bracknell, UK (division of Cawood Scientific Ltd) for soil analysis. Measurements made were: pH, available Phosphorus, Potassium, Magnesium, Ammonium Nitrate, estimated Cation exchange capacity plus extractable Sodium and Calcium. The percentage organic matter from loss on ignition was determined as was the percentages of sand (size range 0.063–2 mm), silt (size range 0.002–0.063 mm) and clay (size range < 0.002 mm) in each sample.

### 2.3 DNA extraction from nematodes

The nematodes were counted under a microscope before pelleting for DNA extraction. The nematode pellet was re-suspended in 100 μl of lysis buffer (100 mM NaCl, 10 mM Tris pH 8, 10 mM EDTA, 1% SDS, 1% β-*mercaptoethanol*, 100 μgml^−1^ proteinase K), incubated at −20 °C for at least 30 min and 60 °C for 1 h. The nematode DNA was then extracted by x2 phenol:chloroform and precipitated with isopropanol. The DNA was re-suspended in nuclease-free water and provided a template for PCR.

### 2.4 PCR of nematode barcode and bioinformatics analyses

PCR amplification of nematode DNA was performed with 0.5 units Phusion DNA Polymerase, 1 μl template DNA, 0.5 μM primers, 200 μM dNTPs and 1× Phusion HF buffer in the following conditions: 98 °c 30 s, (98 °C for 10s, 62 °C for 30 s, 72 °C for 30 s) x30 then 72 °C for 5 min. Primers NF1 (5′-GCCTCCCTCGCGCCATCAGGGTGGTGCATGGCCGTTCTTAGTT-3′; forward) and 18sr2b (5′-GCCTTGCCAGCCCGCTCAGTACAAAGGGCAGGGACGTAAT-3′; reverse) were used to amplify a SSU barcoding region (Porazinska et al., 2009). The products were submitted for AmpliconEZ sequencing (Genewiz) after visualising and verifying the PCR product on a 1% agarose gel. All raw sequence data can be accessed at SRA under submission SUB9102711 (https://submit.ncbi.nlm.nih.gov/subs/sra/SUB9102711).

All sequences were quality checked using FastQC and paired-end reads were joined with the program fastq-join. The resultant fasta sequences were trimmed for quality and chimeric sequences were filtered using DADA2 in the QIIME2 pipeline (Callahan et al., 2016; Bolyen et al., 2019). The pipeline was used to identify the taxonomic composition of the samples using the SILVA 132 95% OTU sequence reference database (Quast et al., 2013). Sequences that were not identified were omitted from analyses. Nematodes were assigned to genus or a higher taxonomic order based on a similarity of at least 95% to entries in the data base as in previous work (Schenk et al., 2020). The relative abundance of each taxonomic unit within a sample that was not a plant-parasitic nematode was calculated based on its allocated percentage of reads.

### 2.5 Nematode community indices

Values were calculated following details provided by Neher et al. (2004) and values for feeding habits (Yeates et al., 1993). The nematode channel ratio is calculated as the percentage of bacterivores relative to their number plus that of the fungivores. The enrichment, structural, basal, and channel indices differ in that their calculation involves a weighing system for nematode functional guilds (Ferris et al., 2001; Berkelmans et al., 2003). The weightings used were the colonizer-persister scale (cp 1–5) from Bongers and Bongers (2001) with additions (Hánĕl and Čerevková, 2010; Fiscus and Neher, 2002; Yan et al., 2018). Trophic diversity is the reciprocal of the three proportions of bacterial, fungal and all other trophic forms each squared. Two standard diversity indices, Shannon Weaver and Simpson were also calculated (Neher et al., 2004).

### 2.6 Statistical analyses

Means are given ± standard error of the mean (S.E.). Data were analysed using univariate and One-way ANOVA, linear regression and cluster analysis in SPSS (SPSS v26; IBM Corporation eArmonk, New York, USA). Comparison of the nine banana plantations established there was no significant difference in nematode abundance 100 g^−1^ soil between the four east and five west locations (*P* = 0.28 *a priori* contrasts, One-way ANOVA) so region was not considered in subsequent analyses. Cluster analysis was based on Ward's method, with the interval based on squared Euclidean distance. Values were transformed to Z scores before computing proximities and measures rescaled to 0–1 range after the distance measure has been computed. The analysis was augmented by R studio (version 3.6.0) using package pvclust for hierarchical clustering.

## 3 Results

### 3.1 Soil analysis

One-way ANOVA established that none of the soil analytical values differed significantly among the samples obtained from the three plantation types. The overall mean values were: pH 6.45 ± 0.07; P (mg/l) 17.8 ± 5.49; K (mg/ml) 234, ± 40.4; Mg (mg/ml) 281 ± 21.3; Na (mg/ml) 12.6 ± 4.04; Ca (mg/ml) 1484 ± 131; Cation exchange (meq/100 g) 15.7 ± 1.12; % sand, 30.9 ± 6.06; % silt, 32.5 ± 3.13; % clay 41.1 ± 3.58 and % organic matter, 6.24 ± 0.49. The values for pH and % organic matter of each soil site are provided in Table 1. All values listed in Supplementary Table S1.

### 3.2 Nematode numbers differ in soil surrounding banana-coffee plants

The soil nematodes present in the fresh soil samples obtained from banana, coffee and banana-coffee fields ranged from 200 to 1100 nematodes 100 g^−1^ soil with a mean of 337 ± 35, 553 ± 126 and 893 ± 121 respectively (Table 1). Comparison after logarithmic transformation established that the mean nematodes g^−1^ soil differed for the three crops (Table 4; *P* < 0.05; Student–Newman–Keuls, One-way ANOVA).

### 3.3 Nematodes identified in each sample

The taxa detected in each sample are given in Table 2. The frequency of detected presence varied from *Aphelenchoides* being present in all 15 samples to *Alaimus*, *Oscheius*, *Rhabdolaimus*, *Mesodorylaimus* and *Ditylenchus* only being detected in a single sample.

**Table 2:** The number of sampled Ugandan fields of three plantation types where each nematode taxa was detected, along with their cp values.

Nematode taxon	Banana (/9 fields)^a^	Banana-coffee (/3 fields)^a^	Coffee (/3 fields)^a^	c-p scale (1–5)
Bacterivores (Ba)				
*Diplogaster*	2	ND	ND	1
Diplogasterida	1	ND	3	1
*Diploscaper*	1	3	ND	1
*Monhystera*	8	3	2	1
*Oscheius*	1	ND	ND	1
*Panagrolaimus*	2	ND	2	1
*Rhabditis*	8	3	1	1
*Brevibucca*	4	1	1	2
*Cephalobus*	7	3	3	2
*Cervidellus*	2	ND	ND	2
*Geomonhystera*	3	3	ND	2
Araeolaimida	8	2	3	3
Chromadorida	8	1	ND	3
*Desmolaimus*	6	2	2	3
*Prismatolaimus*^b^	4	ND	3	3
*Rhabdolaimus*	ND	ND	1	3
Alaimida	3	3	ND	4
*Alaimus*	1	ND	ND	4

Fungivores (Fu)				
*Aphelenchoides*	9	3	3	2
*Aphelenchus*	3	3	2	2
*Ditylenchus*	1	ND	ND	2
*Nothotylenchus*	1	1	ND	2
*Pseudoacrobeles*	3	3	3	2

Predators (Pr)				
Mononchida	4	ND	1	4
*Aporcelaimellus (also Om)*	1	1	ND	5

Omnivores (Om)				
*Dorylaimoides*	2	ND	1	4
*Dorylaimus*	7	1	3	4
*Eudorylaimus*	3	ND	ND	4
*Mesodorylaimus*^b^	1	ND	ND	5

a ND, the nematode was not detected in any of the samples from a plantation type. The feeding type for each taxon is designated as Bacterivore (Ba), Fungivore (Fu), Predator (Pr) and Omnivore (Om).

b These genera may be predators (Wang et al., 2015).

### 3.4 Plantation specific profiles of soil nematodes

**Fig. 1:**
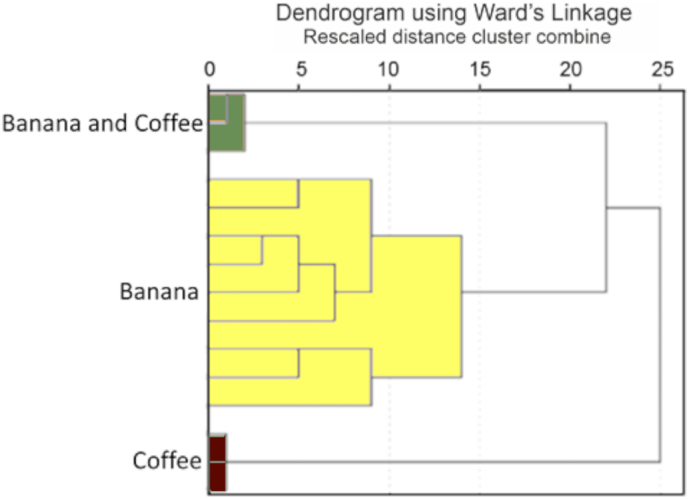
A hierarchical cluster dendrogram based on abundance of the identified nematode taxa in banana, banana-coffee intercrop and coffee plantations. The three plantations types belong to statistically significant clusters (*P* < 0.05, multiscale bootstrap resampling).

Cluster analysis was carried out to explore the relationships among the 15 samples. Discriminating the samples by abundance of identified nematode taxa yielded three clusters (P < 0.05, multiscale bootstrap resampling). These clusters represented, without exception, each of the three plantation types (Fig. 1). The basis of the discrimination by cluster analysis of the three planation types was examined by comparing differences in the abundance of the identified nematode taxa.

A total of nine taxons showed significant differences in mean abundance between crops (*P* < 0.05; Student–Newman–Keuls, One-way ANOVA; Table 3). Increases in abundance between plantation types were often >5 fold. Diplogasterida (Ba1), Araeolaimida, Pristmatolaimus and *Rhabdolaimus* (all Ba3) were most abundant in soil from coffee plantations and *Diploscaper* (Ba1), *Monhystera* (Ba1), *Geomonhystera* (Ba2), and Alaimida (Ba4) for the soil from intercrop plantations. The only fungivore to differ was a greater abundance of *Aphelenchus* (Fu2) in the intercrop plantation. The 15 samples clustered by the crop(s) growing at each plantation, without exception, due to the considerable differences in the number of taxonomic units between samples.

**Table 3:** The soil nematode taxa that differ in their relative abundance by plantation type. Values are means ± S.E. of the percentage of diagnostic sequencing reads per sample. Letters denote significant differences (P < 0.05 based on arc sine transformed values, Student–Newman–Keuls, One-way ANOVA). ND is not detected in the soil samples from that plantation type.

Nematode taxon	Banana	Coffee	Banana & coffee
Alaimida	3.0 ± 1.6^a,b^	ND^b^	7.9 ± 0.4^a^
*Aphelenchus*	2.8 ± 1.8^b^	2.3 ± 0.5^b^	24.5 ± 2.3^a^
Araeolaimida	6.3 ± 1.1^b^	29.3 ± 6.1^a^	2.6 ± 1.9^b^
Diplogasterida	0.5 ± 0.5^b^	8.2 ± 0.8^a^	ND^b^
*Diploscaper*	0.8 ± 0.8^b^	ND^b^	12.6 ± 0.2^a^
*Geomonhystera*	2.0 ± 1.1^b^	ND^b^	6.3 ± 0.3^a^
*Monhystera*	2.9 ± 0.9^b^	1.8 ± 1.4^b^	11.26 ± 0.9^a^
*Prismatolaimus*	3.8 ± 1.7^a,b^	8.2 ± 0 8^a^	ND^b^
*Rhabdolaimus*	ND^b^	6.9 ± 0.6^a^	ND^b^

### 3.5 Nematode community indices

**Fig. 2:**
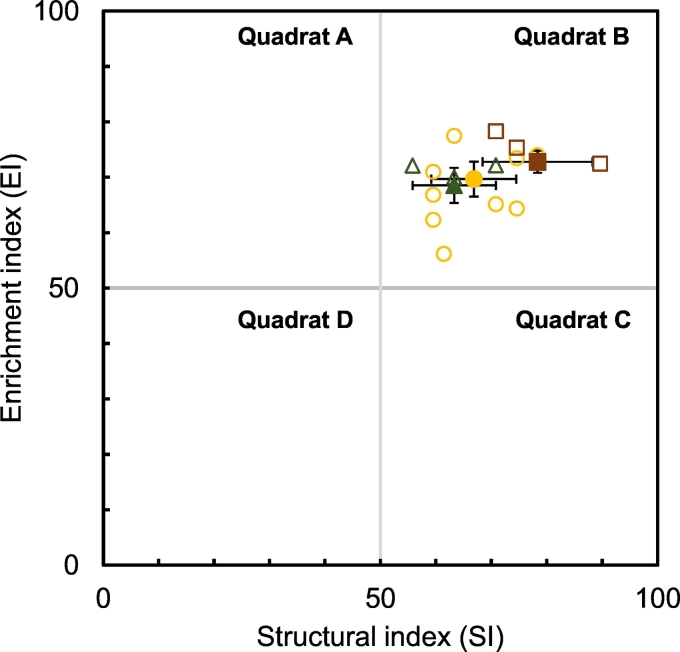
The adjusted enrichment and structural indices for the 15 samples are plotted for banana (circle), banana-coffee interplant (triangle) and coffee (square) with the values for their means ± sample standard deviations (filled symbols).

All fifteen plantations were placed in quadrat B of the plot of enrichment and structural indices (Fig. 2) with no significant differences among the plantation types. This indicates a considerable concordance among the sites. Analysis of the possible influence of soil factors and plantation age on the enrichment index and structural index indicated two significant relationships. Linear regression analysis established that the age of plantation affected the structural index as did the percentage clay content on the enrichment index (both P < 0.05, F test). Multiple regression with a second variable of percentage clay content reached statistical significance for just the enrichment index and with age for only the structural index. Details of the two significant relationships are given in Supplementary data (Fig. S1). Adjustment using the regression equation reduced the coefficient of variation for enrichment index from 8.6% to 4.4% and from 25.6 to 13.4% for structural index. The grand means for the two indices were unaltered. The adjusted values and variance are given to improve precision in Fig. 2 and Table 4.

**Table 4:** The mean number of nematodes per sample and a range of descriptive indices for the banana, coffee and banana-coffee intercrop plantations. Values are means ± S.E. and letters denote significant differences (P < 0.05, Student–Newman–Keuls, One-way ANOVA).

	Banana	Coffee	Banana & Coffee
Nematodes 100 g^−1^ soil	336 ± 35.1^c^	893 ± 121^a^	553 ± 126^b^
Number of taxons	12 ± 0.41^a^	14 ± 0.577^a^	12 ± 1.15^a^
Structural index† (%)	69.01 ± 2.69^a^	79.70 ± 4.31^a^	65.25 ± 5.00^a^
Enrichment index‡ (%)	69.68 ± 1.05^a^	72.79 ± 0.90^a^	68.54 ± 1.83^a^
Basal index (%)	17.48 ± 2.44^ab^	10.31 ± 0.53^b^	23.36 ± 0.41^a^
Channel index	35.52 ± 3.73^a^	27.79 ± 2.18^a^	28.07 ± 0.70^a^
Nematode channel ratio	0.588 ± 0.032^a^	0.713 ± 0.018^a^	0.570 ± 0.010^a^
Shannon Weaver index	2.13 ± 0.063^a^	2.25 ± 0.086^a^	2.22 ± 0.082^a^
Simpson index	0.136 ± 0.021^a^	0.148 ± 0.024^a^	0.130 ± 0.012^a^
Trophic diversity	2.30 ± 0.058^a^	1.70 ± 0.043^c^	2.03 ± 0.045^b^
Cp value 1 (%)	15.2 ± 1.86^b^	15.1 ± 2.78^b^	27.1 ± 0.79^a^

† Adjusted for the significant effect of plantation age.

‡ Adjusted for the significant effect of % clay in the soil samples.

Only the basal and trophic diversity indices and the proportion of nematodes that are rapid colonisers (cp-1) revealed differences in values among the three plantation types (Table 4). The soils of the banana-coffee intercrop had a higher percentage of rapid colonisers (cp-1). The intercrop had the highest basal index with a value that was significantly greater than in the coffee only plantations. The values for all the descriptive indices are given in Table 4. Overall means did not change with plantation type for structural, enrichment, channel, Shannon-Weaver or Simpson indices. The number of taxons/sample and the nematode channel ratio also did not differ with the plantation type.

## 4 Discussion

The range of nematode densities in the 15 samples from 11 locations spanning 250 km was consistent with estimates for Uganda within a global scale study (van den Hoogen et al., 2019). Values in quadrat B for all plantations sampled in Fig. 2 indicate a mature soil web condition with a low physical disturbance, limited chemical stressors, a high nutrient enrichment and balanced decomposition channels (Ferris et al., 2012). This similarity among the sites facilitates the detection of any major consequences from any future changes in cropping or agronomy. One difference was that the three types of plantations had dissimilar nematode densities. Analysis clustered the soil nematode community correctly by plantation type confirming greater differences among them than among the banana plantations. Clusters were only revealed when classifying samples by abundance of taxa and not merely by their detected presence or absence. This indicates that nematode population densities are differentially modulated in response to the crop(s) grown. Knowledge of the bases for niche separation is too incomplete for soil nematodes to explain the differences in abundance of many taxons among the plantation types although the relationship between body diameter and available pore size plus foraging strategy are likely to be factors (Neher, 2010). The significant negative relationship between the enrichment index and percentage clay in the soil samples may relate to the available pore space for the nematodes. Nematode movement is dependent in most soils on larger soil particles than provided by clay (Jones et al., 1969). A similar effect was evident for the structural index but it did not reach statistical significance. The positive relationship between the structural index and the age of the plantations is consistent with a low level of soil disturbance and a maturing of the food web with increased involvement of species with relatively long life cycles (Ferris et al., 2001).

The only significant differences in indices among the three plantations were for the trophic diversity and basal indices and the proportion of cp-1 nematodes. An increase in trophic diversity indicates a greater occurrence of less abundant trophic groups (Neher et al., 2004). The highest trophic diversity was associated with soils supporting banana with an intermediate value for the intercrop and lowest for coffee only plantations. The basal index indicates the abundance of general opportunists (Minoshima et al., 2007). The grand mean channel index value for the three types of plantations (32.48 ± 2.43%) indicates both fungivores and bacterivores contributed appreciably to the enrichment index. The fungal pathways involve a slow decomposition rate of more complex materials than utilised by bacterivores (Ferris and Matute, 2003). This is consistent with the use of mulches and stover rather than inorganic fertilisers.

The enrichment index was similar for the three cropping regimes but 2.6× more soil nematodes 100 g^−1^ soil were associated with the coffee than the banana crop. This greater biomass ensures a greater contribution to crop nutrition from nematode excretion which enhances the availability of net phosphate (Irshad et al., 2011; Gebremikael et al., 2016) and possibly other substances for which deficiencies do occur (van Asten et al., 2003).

A lower enrichment index than observed in the Ugandan banana plantations was reported for soil nematodes associated with dessert banana monoculture plantations in both Australia (Pattison et al., 2008) and Hainan Island, China (Zhong et al., 2016) based on morphological assessments. The structural index was also lower for the data collected from China compared to Uganda. The main limitation to such comparisons is the possible effect of very different environments and planting materials. If that caveat is set aside, all the Ugandan cooking banana plantations had similar enrichment and structural indices to those reported in Australia for conventional but not organic banana production which had a lower enrichment index. The nematode soil community associated with monoculture, dessert banana in China was characterised by low enrichment and structural indices with only a small effect of annual tillage on that crop. All nine Uganda banana plantations had a channel ratio that indicated balanced decomposition processes as occurred for organic but not conventional production in Australia. A balanced decomposition pattern also prevailed when plant residues rather than less complex materials were used as fertiliser for dessert banana in Guadeloupe (Tabarant et al., 2011). The diversity of nematodes in the Ugandan plantations were greater *i.e.* lower Simpson index values than the corresponding means in Australia for either the conventional or organic plantations. A slightly greater Shannon Weaver index reflecting increasing species dominance, and richness occurred for banana plantations in Uganda than for conventional and organic plantations in Australia.

The values for enrichment and structural indices of the three Ugandan coffee plantations were similar to those at two sites in Brazil when organic rather than chemical fertilisers were applied although a further three organic sites had lower structural indices (Lujan Soto and Lopes Vieira, 2018). Favourable enrichment and structural indices are important for Uganda as its small-scale farmers gain 21%–85% of total income from their coffee harvest. Coffee represents Uganda's most valuable annual export ($490 million) so soil management of its plantations is important (Bongers et al., 2015).

The lack in change of the nematode community with the duration of the banana plantation contrasts with a strawberry replant problem that occurs after its long-term production in one soil. This caused a decline in the Shannon-Weaver, channel and basal indices and the nematode channel ratio over a seven-year period that is related to its roots secreting phenolic acids (Li et al., 2016). No comparable significant decline for any of these indicators were detected with age of the banana plantations in the current work. Monoculture of maize and soybean also results in a declining yield in part due to biotic factors as reflected by changes in fungal communities (Strom et al., 2020). Any future use of certain pesticides is also likely to alter the nematode soil community as detected in previous work with nematicide application to a potato crop (Celis et al., 2004).

Indicator species for different source stresses have been reported but are unlikely to be present in all Ugandan soils. Consequently, community level values such as the structural and basal indices provide a more universally applicable approach for soil. It would be of value to determine if soil quality declines with maize given that the area planted with this crop is increasing in Uganda (FAOSTAT, 2020) particularly as high soil erosion rates are also likely to occur where maize is grown (Lufafa et al., 2003). Any benefit of short term replacement of banana by maize would not be advantageous if as a consequence there was a long-term detriment to the soil.

Effort to improve the quality of soils to enhance yields of a crop such as banana requires baseline data. Biological indicators are key to providing this in ways other indicators are not because the majority of soil processes are intrinsically linked to soil biota (Stone et al., 2016). The potential of nematodes for such work in Uganda led to initial work on PCR-based analysis of 18S rRNA gene sequences (Nakacwa et al., 2013). Next generation sequencing supersedes that approach and has been applied to *Plasmodium falciparum* in Uganda (Boyce et al., 2018) and the genomes of plant viruses in Africa including Uganda (Ibaba and Gubba, 2020). Implementation of the approach for soil quality in Uganda is likely to be incremental. At first, progress can be made with costs and necessary expertise in Uganda limited to collecting soil samples for sequencing and analysis by international partners, as in the current work. Next generation sequencing is already carried out by several institutes with a presence in Africa that collaborate with Ugandan scientists. They, or other international partners, could support the training of Ugandan scientists for the next step of in-country analysis of sequence information. There is considerable interest in developing capacity in next generation sequence surveillance of human pathogens in Africa (Inzaule et al., 2021) that is likely in the longer-term to enhance capacity for other applications such as monitoring soil quality. The importance of banana for food security in Uganda is likely to attract international donors and any necessary international collaboration for next-generation sequencing to underpin yield enhancement. Defining where soils of poor soil quality occur in Uganda is of value given many are currently inadequate (van Asten et al., 2003; Wortmann and Kaizzi, 1998).

## 5 Conclusions

This work identified crop-specific soil nematode communities, indicating that the population is differentially modulated in response to the crop choice, and established that nematode soil community analysis has the potential to assess soil quality of banana plantations in Uganda. It suggests that these plantations benefit from desirable nematode community services. Assessment of the community could provide useful sentinels in future for reporting optimal use of organic fertilisers and the consequences of changes in agronomy or crop. The approach used is capable of a high throughput and could provide a means of ensuring banana and other crops contribute optimally to the future food security of Uganda.

## Declaration of competing interest

The authors declare that they have no known competing financial interests or personal relationships that could have appeared to influence the work reported in this paper.

## Appendix A Supplementary data

Supplementary material Image 1
