# Supplementary material for: Next-generation sequencing of the soil nematode community enables the sustainability of banana plantations to be monitored
Source: Appl Soil Ecol. 2021 Oct;166:None. doi: 10.1016/j.apsoil.2021.103999 (PMC8326924; doi:10.1016/j.apsoil.2021.103999)
Supplement: Supplementary file 1 — Supplementary material [file mmc1.docx]

**Next-generation sequencing of the soil nematode community enables the sustainability of banana plantations to be monitored**

**Supplementary Material**

Christopher A. Bell^a^, Josephine Namaganda^b^, Peter E. Urwin^a*^ and Howard J. Atkinson^a^

^a^Centre for Plant Sciences, School of Biology, University of Leeds, Leeds, UK

^b^National Agriculture Research Laboratories, Kampala, Uganda

Table S1. Soil analysis data for the banana, coffee and banana/coffee plantations.

| **Field** | **Crop** | **pH** | **P (mg/l)** | **K (mg/l)** | **Mg (mg/l)** | | | **Na (mg/l)** | **Ca (mg/l)** | | **Cation exchange (meq/100g)** | **Sand (%)** | **Silt (%)** | **Clay (%)** | **Organic Matter (%)** |
| --- | --- | --- | --- | --- | --- | --- | --- | --- | --- | --- | --- | --- | --- | --- | --- |
| 1 | Banana | 6.1 | 2.6 | 92 | | 189 | 5.7 | | | 1016 | 12.3 | 49 | 49 | 30 | 4.4 |
| 3 | Banana | 6.4 | 5.4 | 137 | | 224 | 5.0 | | | 1194 | 12.9 | 53 | 53 | 28 | 5.0 |
| 4 | Banana | 6.6 | 13.2 | 120 | | 240 | 4.8 | | | 1442 | 14.1 | 55 | 16 | 29 | 5.2 |
| 5 | Banana | 6.6 | 6.4 | 72 | | 190 | 6.3 | | | 1105 | 11.1 | 45 | 23 | 32 | 4.4 |
| 6 | Banana | 6.5 | 77.2 | 541 | | 323 | 9.1 | | | 1779 | 18.9 | 5 | 43 | 52 | 8.3 |
| 8 | Banana | 6.2 | 51.6 | 419 | | 429 | 21.5 | | | 1857 | 21.6 | 4 | 37 | 59 | 8.2 |
| 9 | Banana | 6.5 | 4.0 | 116 | | 281 | 4.9 | | | 1366 | 14.4 | 22 | 34 | 44 | 5.3 |
| 10 | Banana | 6.7 | 22.4 | 284 | | 352 | 66.8 | | | 2943 | 25.7 | 9 | 45 | 46 | 9.5 |
| 11 | Banana | 6.8 | 33.0 | 172 | | 316 | 9.4 | | | 1866 | 17.0 | 4 | 30 | 66 | 7.9 |
|  | **Mean ± S.E.** | **6.49 ±** 0.075 | **24.0 ±** 8.6 | **217 ±** 54.6 | | **283 ±** 26.8 | **14.8 ±** 6.7 | | | **1619 ±** 197 | **16.4 ±**  1.6 | **27.3 ±** 7.6 | **36.7 ±** 4.1 | **42.9 ±** 4.7 | **6.5 ±**  0.7 |
| 2 | Coffee | 6.2 | 3.8 | 168 | | 210 | 7.3 | | | 932 | 12.0 | 69 | 17 | 21 | 4.1 |
| 4 | Coffee | 6.9 | 5.4 | 404 | | 239 | 8.6 | | | 1145 | 12.2 | 46 | 21 | 33 | 4.6 |
| 7 | Coffee | 6.6 | 4.2 | 54 | | 196 | 5.0 | | | 1197 | 11.6 | 48 | 19 | 33 | 5.4 |
|  | **Mean ± S.E.** | **6.57 ±** 0.20 | **4.5 ±** 0.48 | **209 ±** 103 | | **215 ±**12.7 | **7.0 ±** 1.05 | | | **1091 ±** 81.1 | **11.9 ±**  0.18 | **54.3 ±** 7.36 | **19.0 ±** 1.15 | **29.0 ±** 4.00 | **4.7 ±**  0.38 |
| 7 | Banana-coffee | 6.3 | 18.2 | 471 | | 386 | 12.9 | | | 1616 | 19.3 | 5 | 36 | 59 | 8.5 |
| 9 | Banana-coffee | 6.1 | 12.0 | 270 | | 408 | 13.3 | | | 1604 | 19.2 | 6 | 41 | 53 | 8.0 |
| 10 | Banana-coffee | 6.3 | 7.6 | 184 | | 228 | 8.8 | | | 1198 | 13.5 | 44 | 24 | 32 | 4.8 |
|  | **Mean ± S.E.** | **6.23 ±** 0.067 | **12.6 ±** 3.07 | **308 ±** 85.0 | | **341 ±** 56.7 | **11.7 ±** 1.44 | | | **1473 ±** 137 | **17.3 ±**  1.92 | **18.3 ±** 12.8 | **33.7 ±** 5.04 | **48.0 ±** 8.19 | **7.1 ±**  1.16 |
| **Grand Mean ±** | | **6.45 ±** | **17.8^†^ ±** | **234 ±** | | **281 ±** | **12.6 ±** | | | **1484 ±** | **15.7 ±** | **30.9 ±** | **32.5 ±** | **41.1 ±** | **6.24^†^ ±** |
|  | **S.E.** | 0.065 | 5.49 | 40.42 | | 21.34 | 4.04 | | | 130.55 | 1.12 | 6.06 | 3.13 | 3.58 | 0.49 |
| **ANOVA P Value** | | 0.221 | 0.373 | 0.683 | | 0.180 | 0.774 | | | 0.315 | 0.243 | 0.128 | 0.080 | 0.214 | 0.27 |

^†^, the data for this variable lacks homogenous variance (P<0.05, Levene statistic).

Fig S1. Regression of the enrichment index (EI) with percentage clay in each soil sample and structural index (SI) with age of the planation in the fifteen soil samples from plantations of banana (circles), coffee (squares) and banana and coffee interplants (triangles).

The regression equations are: enrichment index = - 0.2237 x % clay + 79.276 and structural index = 3.7601 x age + 52.043. The regression lines are statistically significant (P<0.05 in both cases). Adding plantation age to a multiple linear regression for enrichment index and % clay to that for structural index did not result in statistical significance for those steps and so the two multiple regressions were rejected. The equations given were used to adjust values for each soil sample in Table 4 and Fig. 2.
